# Crystal structure of μ-oxalodi­hydroxamato-bis­[(2,2′-bipyrid­yl)(di­methyl sulfoxide-κ*O*)copper(II)] bis­(perchlorate)

**DOI:** 10.1107/S2056989016000050

**Published:** 2016-01-13

**Authors:** Irina A. Odarich, Anna V. Pavlishchuk, Valentina A. Kalibabchuk, Matti Haukka

**Affiliations:** a O.O. Bohomolets National Medical University, Department of General Chemistry, Pr. Pobedy, 34, Kiev, 03055 , Ukraine; b Taras Shevchenko National University of Kiev, Department of Chemistry, Volodymyrska str. 62, Kiev, 01601 , Ukraine; c University of Joensuu, Department of Chemistry, PO Box 111, FI-80101 Joensuu, Finland

**Keywords:** crystal structure, Cu(II) complex, oxalodi­hydroxamic acid

## Abstract

The centrosymmetric binuclear complex, [Cu_2_(C_2_H_2_N_2_O_4_)(C_10_H_8_N_2_)_2_(C_2_H_6_OS)_2_](ClO_4_)_2_, contains two copper(II) ions, connected through an N-deprotonated oxalodi­hydroxamic acid dianion, two terminal 2,2′-bi­pyridine ligands, and two apically coordinating dimethylsulfoxide mol­ecules. Two non-coordinating perchlorate anions assure electrical neutrality. The copper(II) ions in the complex dication [Cu_2_(C_10_H_8_N_2_)_2_(μ-C_2_H_2_N_2_O_4_)(C_2_H_6_SO)_2_]^2+^ are in an O_2_N_3_ square-pyramidal donor environment, the Cu–Cu separation being 5.2949 (4) Å. Two hydroxamate groups in the deprotonated oxalodi­hydroxamic acid are located *trans* to one each other. In the crystal, O—H⋯O and C—H⋯O hydrogen bonds link the complex cations to the perchlorate anions. Further C—H⋯O hydrogen bonds combine with π–π contacts with a centroid-to-centroid separation of 3.6371 (12) Å to stack the mol­ecules along the *a*-axis direction.

## Abstract 2

In this article we report a synthetic procedure and structure of the novel dinuclear copper(II) complex, with a bridging oxalodi­hydroxamate ligand and terminal 2,2′-bi­pyridine and DMSO ligands completing the square pyramidal coordination spheres of the Cu(II) centres..

## Chemical context   

Syntheses of complexes based on functionalized hydroxamic acids are of particular inter­est due to their non-trivial magnetic (Pavlishchuk *et al.*, 2014 ▸) and luminescence (Jankolovits *et al.*, 2011 ▸) properties, potential applications in bioinorganic modeling (Marmion *et al.*, 2004 ▸), adsorption (Pavlishchuk *et al.*, 2010 ▸, 2011*a* ▸;), catalysis (Mezei *et al.*, 2007 ▸) and the creation of recognition agents (Lim *et al.*, 2011 ▸). The majority of complexes obtained with hydroxamic acids and additional donor ligands belong to different families of metallacrown coordination compounds (Mezei *et al.*, 2007 ▸). Other topologies for polydentate hydroxamate-based complexes are more unusual (Gumienna-Kontecka *et al.*, 2013 ▸; Golenya *et al.*, 2014 ▸). Here we present the structure of the binuclear complex [Cu_2_(C_10_H_8_N_2_)_2_(μ-C_2_H_2_N_2_O_4_)(C_2_H_6_SO)_2_](ClO_4_)_2_ (I), obtained from oxalodi­hydroxamic acid and bi­pyridine in DMSO solution.
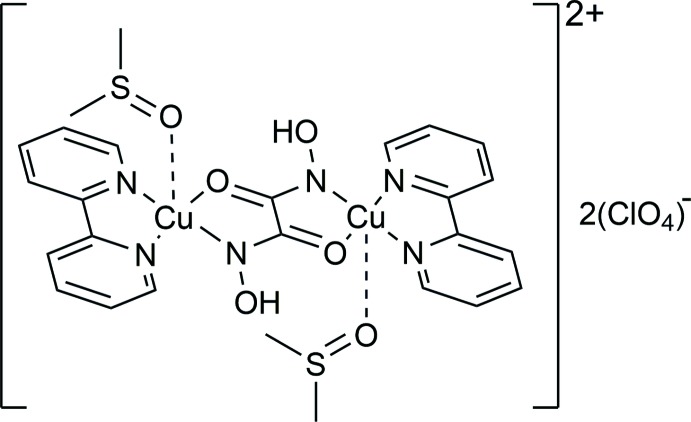


## Structural commentary   

**Figure 1:**
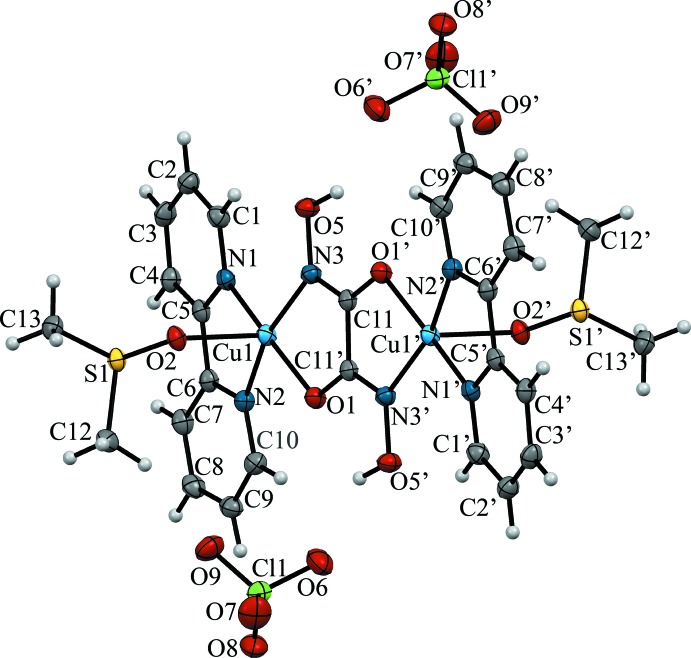
The crystal structure of complex (I), showing the atom labeling. Displacement ellipsoids are drawn at the 50% probability level.

The title compound (I) consists of a centrosymmetric complex di-cation [Cu_2_(C_10_H_8_N_2_)_2_(μ-C_2_H_2_N_2_O_4_)(C_2_H_6_SO)_2_]^2+^ with two uncoordinating perchlorate counter-anions (Fig. 1 ▸). The two copper(II) cations are connected through a doubly deprotonated oxalodi­hydroxamic acid, which serves as a bridging ligand between the copper ions which are coordinated by two nitro­gen atoms from the 2,2′-bi­pyridine ligand, one carbonyl oxygen atom and the deprotonated hydroxamate nitro­gen atom from one half of the oxalodi­hydroxamato ligand and the O atom of a DMSO mol­ecule. The oxalodi­hydroxamato dianion is in a *trans*-form, while for metallacrown formation the *cis*-form is preferred. The coordination sphere of the copper(II) cation is square-pyramidal (τ = 0.21; Addison *et al.*, 1984 ▸) and the copper(II) ion deviates from the mean plane of the O1/N1/N2/N3 donor atoms by 0.1868 (2) Å. The separation between the copper (II) cations is 5.2949 (4) Å. The equatorial Cu—N and Cu—O distances are typical of those for copper(II) complexes with hydroxamate and oxime donor groups (Buvailo *et al.*, 2012 ▸; Duda *et al.*, 1997 ▸; Pavlishchuk *et al.*, 2011*b* ▸; Safyanova *et al.*, 2015 ▸, Table 1 ▸). The elongated apical bond, Cu1—O2 (2.2516 (16) Å), compared to the Cu—O and Cu—N distances in the equatorial plane that range from 1.9848 (16) to 1.9966 (19) Å, Table 1 ▸, is most likely due to Jahn–Teller distortion.

The C—N and C—C bond lengths in the 2,2′-bi­pyridine ligands are also normal for 2-substituted pyridine derivatives (Krämer *et al.*, 2000 ▸; Strotmeyer *et al.*, 2003 ▸; Fritsky *et al.*, 2004 ▸). The coordinating oxalo­hydroxamate dianion also has C—C, C—N, N—N bond lengths that are typical of N-deprotonated hydroxamate groups (Świątek-Kozłowska *et al.*, 2000 ▸; Dobosz *et al.*, 1999 ▸).

## Supra­molecular features   

**Figure 2:**
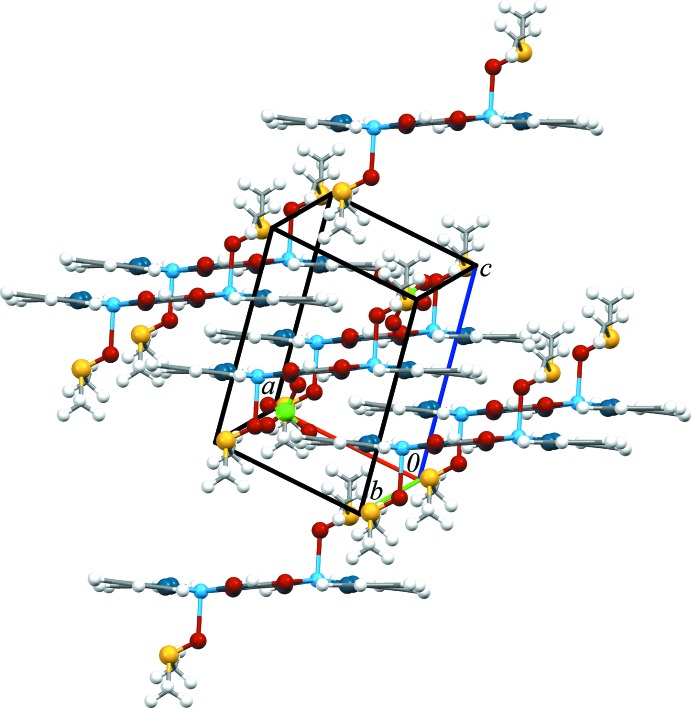
The crystal packing of complex (I).

In the crystal structure, O5—H5*O*⋯O6 together with C12—H12*A*⋯O9 hydrogen bonds link the cations and associated perchlorate anions. An extensive series of other C—H⋯O contacts, Table 2 ▸, link the complex cations to other anions. The O2 atom of the DMSO ligand acts as a bifurcated acceptor forming C4—H4⋯O2 and C7—H7⋯O2 hydrogen bonds. These hydrogen bonds combine with π–π contacts between the N2/C6–C10 ring of the bi­pyridine and the Cu1/O1/C11/C11^i^/N3 ring formed by the chelating oxalodi­hydroxamate ligand with a centroid-to-centroid distance of 3.6371 (12) Å to stack the cations along the *a-*axis direction, Fig. 2 ▸.

## Database survey   

A search in the Cambridge Structural Database (Version 5.35, May 2014; Groom & Allen, 2014 ▸) shows that there are seven reports devoted to the study of crystal structures of oxalodi­hydroxamic acid and its complexes. In the reported crystal structures of oxalodi­hydroxamic acid and its salts, the compound crystallized only in the *trans*-form. The bond lengths in oxalodi­hydroxamic acid itself and in its ammonium and thallium salts do not differ significantly [C—C bonds are in the range 1.51 (2)–1.528 (3) Å, C=O 1.231 (3)–1.248 (3) Å, C—N 1.310 (4)–1.33 (2) Å while the N—O bond lengths vary from 1.36 (2) to 1.388 (1) Å; Lowe-Ma & Decker, 1986 ▸; Sameena Begum *et al.*, 1987 ▸, 1988 ▸; Huang *et al.*, 1991 ▸; Marsh, 1989 ▸). Only two structures of coordination compounds with di­hydroxy­oxamidato ligands were found. Both involved anionic mononuclear Ni^II^ complexes with ligands derived from doubly or triply deprotonated oxalodi­hydroxamic acid. In one of these complexes (Moroz *et al.*, 2006 ▸), the di­hydroxy­oxamidato trianion acts as a simple bidentate chelating ligand forming a square-planar complex. In the second (Świątek-Kozłowska *et al.*, 2000 ▸), a square planar Ni^II^ complex again forms, but the di­hydroxy­oxamidato ligand also forms bridges to the potassium counter-ions generating a polymeric system. The structure presented here is the first example in which a di­hydroxy­oxamidato anion acts as a bridging ligand between two transition metals. The lack of crystal data for complexes with other transition metal cations may be associated with the ease of hydrolysis of the oxalodi­hydroxamic acid initiated by a metal salt solution.

## Synthesis and crystallization   

To the warm mixture containing 0.060 g (0.5 mmol) of oxalodi­hydroxamic acid and 0.370 g (1 mmol) of Cu(ClO_4_)_2_·6H_2_O in 10 ml of DMSO the solution of 2,2′-bi­pyridine (0.156 g, 1 mmol) in 10 ml of methanol was added upon stirring. The resulted solution was stirred for 1 h and then left for slow evaporation.

The resulting blue crystals suitable for X-ray analysis were isolated after one week. The crystals were washed with small amounts of 2-propanol and dried in air, yielding 0.255 g (28%) of the title compound.

## Refinement   

Crystal data, data collection and structure refinement details are summarized in Table 3 ▸. The OH hydrogen atom was located from a difference Fourier map and was refined isotropically. Other hydrogen atoms were positioned geometrically and were constrained to ride on their parent atoms, with C—H = 0.95–0.98 Å, and *U*_iso_ = 1.2–1.5 *U*_eq_(parent atom). The highest peak is located 0.99 Å from atom Cu1 and the deepest hole is located 0.82 Å from atom Cu1.

## Supplementary Material

Crystal structure: contains datablock(s) I, New_Global_Publ_Block. DOI: 10.1107/S2056989016000050/sj5487sup1.cif

Structure factors: contains datablock(s) I. DOI: 10.1107/S2056989016000050/sj5487Isup2.hkl

CCDC reference: 1445115

Additional supporting information:  crystallographic information; 3D view; checkCIF report

## Figures and Tables

**Table 1:** Selected geometric parameters (Å, °)

Cu1—O1	1.9848 (16)	Cu1—O2	2.2516 (16)
Cu1—N2	1.985 (2)	O1—C11	1.286 (3)
Cu1—N3^i^	1.986 (2)	O5—N3	1.404 (3)
Cu1—N1	1.9966 (19)		
			
O1—Cu1—N2	90.36 (7)	O1—Cu1—O2	98.04 (6)
O1—Cu1—N3^i^	82.73 (7)	N2—Cu1—O2	97.53 (7)
N2—Cu1—N1	81.76 (8)	N3^i^—Cu1—O2	96.15 (7)
N3^i^—Cu1—N1	103.13 (8)	N1—Cu1—O2	90.72 (7)

Symmetry code: (i) 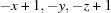.

**Table 2:** Hydrogen-bond geometry (Å, °)

*D*—H⋯*A*	*D*—H	H⋯*A*	*D*⋯*A*	*D*—H⋯*A*
O5—H5*O*⋯O6	0.92 (5)	2.12 (5)	2.912 (3)	144 (4)
C4—H4⋯O2^ii^	0.95	2.42	3.359 (3)	171
C7—H7⋯O2^ii^	0.95	2.31	3.226 (3)	162
C3—H3⋯O7^iii^	0.95	2.50	3.239 (3)	134
C13—H13*A*⋯O7^iv^	0.98	2.56	3.409 (3)	145
C13—H13*C*⋯O8^v^	0.98	2.48	3.346 (3)	148
C13—H13*B*⋯O8^vi^	0.98	2.65	3.442 (3)	138
C12—H12*A*⋯O9	0.98	2.36	3.175 (3)	140
C8—H8⋯O9^ii^	0.95	2.56	3.462 (3)	159
C12—H12*B*⋯O9^vi^	0.98	2.59	3.470 (3)	150

Symmetry codes: (ii) 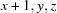; (iii) 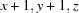; (iv) 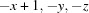; (v) 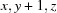; (vi) 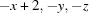.

**Table 3:** Experimental details

Crystal data	
Chemical formula	[Cu_2_(C_2_H_2_N_2_O_4_)(C_10_H_8_N_2_)_2_(C_2_H_6_OS)_2_](ClO_4_)_2_
*M*_r_	912.66
Crystal system, space group	Triclinic, *P* 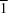
Temperature (K)	100
*a*, *b*, *c* (Å)	7.3641 (2), 10.3759 (5), 12.1358 (5)
α, β, γ (°)	68.853 (2), 84.803 (3), 87.825 (3)
*V* (Å^3^)	861.27 (6)
*Z*	1
Radiation type	Mo *K*α
μ (mm^−1^)	1.59
Crystal size (mm)	0.13 × 0.12 × 0.12

Data collection	
Diffractometer	Nonius KappaCCD
Absorption correction	Multi-scan (*SORTAV*; Blessing, 1995 ▸)
*T*_min_, *T*_max_	0.789, 0.835
No. of measured, independent and observed [*I* > 2σ(*I*)] reflections	18205, 3943, 3351
*R*_int_	0.039
(sin θ/λ)_max_ (Å^−1^)	0.649

Refinement	
*R*[*F* ^2^ > 2σ(*F* ^2^)], *wR*(*F* ^2^), *S*	0.034, 0.087, 1.11
No. of reflections	3943
No. of parameters	241
H-atom treatment	H atoms treated by a mixture of independent and constrained refinement
Δρ_max_, Δρ_min_ (e Å^−3^)	0.74, −0.55

Computer programs: *COLLECT* (Bruker, 2004 ▸), *DENZO*/*SCALEPACK* (Otwinowski & Minor, 1997 ▸), *SHELXS97* (Sheldrick, 2008 ▸), *SHELXL2014*/7 (Sheldrick, 2015 ▸), *Mercury* (Macrae *et al.*, 2008 ▸) and *publCIF* (Westrip, 2010 ▸).

## supplementary crystallographic information

### Crystal data

**Table:**

[Cu_2_(C_2_H_2_N_2_O_4_)(C_10_H_8_N_2_)_2_(C_2_H_6_OS)_2_](ClO_4_)_2_	*Z* = 1
*M**_r_* = 912.66	*F*(000) = 464
Triclinic, *P*1	*D*_x_ = 1.760 Mg m^−^^3^
*a* = 7.3641 (2) Å	Mo *K*α radiation, λ = 0.71069 Å
*b* = 10.3759 (5) Å	Cell parameters from 26719 reflections
*c* = 12.1358 (5) Å	θ = 1.0–27.5°
α = 68.853 (2)°	µ = 1.59 mm^−^^1^
β = 84.803 (3)°	*T* = 100 K
γ = 87.825 (3)°	Block, pale blue
*V* = 861.27 (6) Å^3^	0.13 × 0.12 × 0.12 mm

### Data collection

**Table:**

Nonius KappaCCD diffractometer	3351 reflections with *I* > 2σ(*I*)
Radiation source: fine-focus sealed tube	*R*_int_ = 0.039
ω scans	θ_max_ = 27.5°, θ_min_ = 2.8°
Absorption correction: multi-scan (*SORTAV*; Blessing, 1995)	*h* = −8→9
*T*_min_ = 0.789, *T*_max_ = 0.835	*k* = −13→13
18205 measured reflections	*l* = −15→15
3943 independent reflections	

### Refinement

**Table:**

Refinement on *F*^2^	0 restraints
Least-squares matrix: full	Hydrogen site location: mixed
*R*[*F*^2^ > 2σ(*F*^2^)] = 0.034	H atoms treated by a mixture of independent and constrained refinement
*wR*(*F*^2^) = 0.087	*w* = 1/[σ^2^(*F*_o_^2^) + (0.0388*P*)^2^ + 0.7097*P*] where *P* = (*F*_o_^2^ + 2*F*_c_^2^)/3
*S* = 1.11	(Δ/σ)_max_ < 0.001
3943 reflections	Δρ_max_ = 0.74 e Å^−^^3^
241 parameters	Δρ_min_ = −0.55 e Å^−^^3^

### Special details

**Table:**

Geometry. All e.s.d.'s (except the e.s.d. in the dihedral angle between two l.s. planes) are estimated using the full covariance matrix. The cell e.s.d.'s are taken into account individually in the estimation of e.s.d.'s in distances, angles and torsion angles; correlations between e.s.d.'s in cell parameters are only used when they are defined by crystal symmetry. An approximate (isotropic) treatment of cell e.s.d.'s is used for estimating e.s.d.'s involving l.s. planes.

### Fractional atomic coordinates and isotropic or equivalent isotropic displacement parameters (Å^2^)

**Table:**

	*x*	*y*	*z*	*U*_iso_*/*U*_eq_	
Cu1	0.81268 (4)	0.11133 (3)	0.38671 (2)	0.01840 (10)	
Cl1	0.71265 (8)	−0.30071 (6)	0.19039 (5)	0.02481 (14)	
S1	0.85060 (7)	0.28499 (6)	0.10324 (5)	0.01997 (14)	
O1	0.6724 (2)	−0.05948 (17)	0.41752 (15)	0.0210 (3)	
O2	0.7106 (2)	0.23761 (17)	0.21002 (14)	0.0221 (4)	
O5	0.4262 (2)	−0.26749 (18)	0.48100 (16)	0.0243 (4)	
H5O	0.528 (6)	−0.253 (5)	0.428 (4)	0.083 (14)*	
O6	0.6917 (3)	−0.3371 (2)	0.31798 (18)	0.0383 (5)	
O7	0.5444 (3)	−0.3243 (2)	0.1500 (2)	0.0423 (5)	
O8	0.8557 (3)	−0.3841 (2)	0.16071 (18)	0.0344 (4)	
O9	0.7598 (3)	−0.15753 (19)	0.13534 (19)	0.0365 (5)	
N1	0.9922 (3)	0.2580 (2)	0.36990 (17)	0.0192 (4)	
N2	1.0297 (3)	0.0299 (2)	0.32626 (17)	0.0192 (4)	
N3	0.3959 (3)	−0.1523 (2)	0.51515 (17)	0.0192 (4)	
C1	0.9598 (3)	0.3726 (3)	0.3950 (2)	0.0238 (5)	
H1	0.8409	0.3883	0.4252	0.029*	
C2	1.0948 (3)	0.4694 (3)	0.3781 (2)	0.0255 (5)	
H2	1.0683	0.5499	0.3967	0.031*	
C3	1.2673 (3)	0.4473 (3)	0.3342 (2)	0.0257 (5)	
H3	1.3624	0.5108	0.3248	0.031*	
C4	1.3010 (3)	0.3309 (2)	0.3038 (2)	0.0222 (5)	
H4	1.4180	0.3153	0.2708	0.027*	
C5	1.1604 (3)	0.2381 (2)	0.3224 (2)	0.0199 (5)	
C6	1.1790 (3)	0.1118 (2)	0.2922 (2)	0.0203 (5)	
C7	1.3330 (3)	0.0781 (3)	0.2340 (2)	0.0241 (5)	
H7	1.4356	0.1376	0.2100	0.029*	
C8	1.3359 (3)	−0.0434 (3)	0.2112 (2)	0.0261 (5)	
H8	1.4398	−0.0679	0.1705	0.031*	
C9	1.1844 (3)	−0.1290 (3)	0.2487 (2)	0.0249 (5)	
H9	1.1846	−0.2140	0.2358	0.030*	
C10	1.0341 (3)	−0.0888 (2)	0.3050 (2)	0.0231 (5)	
H10	0.9300	−0.1469	0.3295	0.028*	
C11	0.5220 (3)	−0.0590 (2)	0.4801 (2)	0.0183 (5)	
C12	0.8329 (4)	0.1655 (3)	0.0300 (2)	0.0290 (6)	
H12A	0.8580	0.0719	0.0845	0.044*	
H12B	0.9215	0.1893	−0.0395	0.044*	
H12C	0.7095	0.1696	0.0047	0.044*	
C13	0.7529 (4)	0.4328 (3)	−0.0024 (2)	0.0266 (5)	
H13A	0.6320	0.4095	−0.0173	0.040*	
H13B	0.8315	0.4622	−0.0766	0.040*	
H13C	0.7414	0.5081	0.0286	0.040*	

### Atomic displacement parameters (Å^2^)

**Table:**

	*U*^11^	*U*^22^	*U*^33^	*U*^12^	*U*^13^	*U*^23^
Cu1	0.01496 (15)	0.02100 (16)	0.01911 (16)	−0.00278 (10)	0.00203 (10)	−0.00758 (12)
Cl1	0.0209 (3)	0.0239 (3)	0.0307 (3)	−0.0023 (2)	0.0016 (2)	−0.0117 (2)
S1	0.0164 (3)	0.0234 (3)	0.0179 (3)	−0.0026 (2)	0.0010 (2)	−0.0050 (2)
O1	0.0169 (8)	0.0230 (8)	0.0234 (9)	−0.0041 (6)	0.0043 (6)	−0.0097 (7)
O2	0.0165 (8)	0.0291 (9)	0.0187 (8)	−0.0036 (7)	0.0033 (6)	−0.0069 (7)
O5	0.0229 (9)	0.0232 (9)	0.0297 (10)	−0.0029 (7)	0.0045 (7)	−0.0143 (8)
O6	0.0423 (12)	0.0437 (12)	0.0292 (10)	0.0059 (9)	0.0046 (9)	−0.0157 (9)
O7	0.0267 (10)	0.0498 (13)	0.0519 (13)	−0.0114 (9)	−0.0070 (9)	−0.0181 (11)
O8	0.0354 (11)	0.0327 (10)	0.0356 (11)	0.0075 (8)	0.0041 (8)	−0.0153 (9)
O9	0.0344 (11)	0.0241 (10)	0.0488 (13)	−0.0064 (8)	0.0063 (9)	−0.0120 (9)
N1	0.0182 (9)	0.0218 (10)	0.0167 (9)	−0.0023 (7)	0.0003 (7)	−0.0061 (8)
N2	0.0169 (9)	0.0204 (10)	0.0195 (10)	−0.0008 (7)	−0.0016 (8)	−0.0062 (8)
N3	0.0190 (10)	0.0186 (9)	0.0207 (10)	−0.0015 (7)	0.0004 (8)	−0.0083 (8)
C1	0.0218 (12)	0.0263 (13)	0.0236 (12)	−0.0007 (9)	0.0007 (10)	−0.0099 (10)
C2	0.0274 (13)	0.0210 (12)	0.0271 (13)	−0.0035 (10)	0.0017 (10)	−0.0082 (10)
C3	0.0254 (13)	0.0233 (12)	0.0264 (13)	−0.0074 (10)	−0.0024 (10)	−0.0057 (10)
C4	0.0175 (11)	0.0247 (12)	0.0214 (12)	−0.0029 (9)	0.0001 (9)	−0.0050 (10)
C5	0.0189 (11)	0.0239 (12)	0.0153 (11)	0.0005 (9)	−0.0026 (9)	−0.0050 (9)
C6	0.0179 (11)	0.0229 (12)	0.0187 (11)	−0.0020 (9)	−0.0012 (9)	−0.0058 (9)
C7	0.0173 (11)	0.0294 (13)	0.0236 (13)	−0.0014 (9)	0.0000 (9)	−0.0075 (10)
C8	0.0210 (12)	0.0315 (13)	0.0255 (13)	0.0029 (10)	0.0026 (10)	−0.0113 (11)
C9	0.0264 (13)	0.0253 (13)	0.0248 (13)	0.0029 (10)	−0.0018 (10)	−0.0115 (10)
C10	0.0224 (12)	0.0225 (12)	0.0240 (12)	−0.0004 (9)	−0.0026 (10)	−0.0076 (10)
C11	0.0179 (11)	0.0195 (11)	0.0164 (11)	−0.0001 (9)	−0.0019 (9)	−0.0051 (9)
C12	0.0349 (14)	0.0279 (13)	0.0244 (13)	−0.0011 (11)	0.0054 (11)	−0.0114 (11)
C13	0.0306 (13)	0.0220 (12)	0.0243 (13)	0.0015 (10)	−0.0041 (10)	−0.0046 (10)

### Geometric parameters (Å, º)

**Table:**

Cu1—O1	1.9848 (16)	C2—C3	1.376 (4)
Cu1—N2	1.985 (2)	C2—H2	0.9500
Cu1—N3^i^	1.986 (2)	C3—C4	1.393 (4)
Cu1—N1	1.9966 (19)	C3—H3	0.9500
Cu1—O2	2.2516 (16)	C4—C5	1.388 (3)
Cl1—O9	1.4336 (19)	C4—H4	0.9500
Cl1—O7	1.4339 (19)	C5—C6	1.481 (3)
Cl1—O8	1.4401 (19)	C6—C7	1.382 (3)
Cl1—O6	1.450 (2)	C7—C8	1.384 (4)
S1—O2	1.5234 (17)	C7—H7	0.9500
S1—C12	1.781 (3)	C8—C9	1.390 (4)
S1—C13	1.783 (2)	C8—H8	0.9500
O1—C11	1.286 (3)	C9—C10	1.378 (4)
O5—N3	1.404 (3)	C9—H9	0.9500
O5—H5O	0.92 (5)	C10—H10	0.9500
N1—C1	1.338 (3)	C11—C11^i^	1.486 (5)
N1—C5	1.359 (3)	C12—H12A	0.9800
N2—C10	1.345 (3)	C12—H12B	0.9800
N2—C6	1.355 (3)	C12—H12C	0.9800
N3—C11	1.296 (3)	C13—H13A	0.9800
N3—Cu1^i^	1.986 (2)	C13—H13B	0.9800
C1—C2	1.389 (3)	C13—H13C	0.9800
C1—H1	0.9500		
			
O1—Cu1—N2	90.36 (7)	C2—C3—H3	120.4
O1—Cu1—N3^i^	82.73 (7)	C4—C3—H3	120.4
N2—Cu1—N3^i^	165.41 (8)	C5—C4—C3	118.8 (2)
O1—Cu1—N1	168.93 (7)	C5—C4—H4	120.6
N2—Cu1—N1	81.76 (8)	C3—C4—H4	120.6
N3^i^—Cu1—N1	103.13 (8)	N1—C5—C4	121.6 (2)
O1—Cu1—O2	98.04 (6)	N1—C5—C6	114.7 (2)
N2—Cu1—O2	97.53 (7)	C4—C5—C6	123.7 (2)
N3^i^—Cu1—O2	96.15 (7)	N2—C6—C7	121.8 (2)
N1—Cu1—O2	90.72 (7)	N2—C6—C5	114.2 (2)
O9—Cl1—O7	109.44 (13)	C7—C6—C5	124.1 (2)
O9—Cl1—O8	109.53 (12)	C6—C7—C8	119.1 (2)
O7—Cl1—O8	110.04 (13)	C6—C7—H7	120.4
O9—Cl1—O6	109.19 (13)	C8—C7—H7	120.4
O7—Cl1—O6	109.47 (13)	C7—C8—C9	119.0 (2)
O8—Cl1—O6	109.16 (12)	C7—C8—H8	120.5
O2—S1—C12	105.19 (11)	C9—C8—H8	120.5
O2—S1—C13	105.82 (11)	C10—C9—C8	119.1 (2)
C12—S1—C13	98.84 (13)	C10—C9—H9	120.5
C11—O1—Cu1	110.68 (14)	C8—C9—H9	120.5
S1—O2—Cu1	117.43 (9)	N2—C10—C9	122.2 (2)
N3—O5—H5O	110 (3)	N2—C10—H10	118.9
C1—N1—C5	119.0 (2)	C9—C10—H10	118.9
C1—N1—Cu1	126.77 (16)	O1—C11—N3	127.6 (2)
C5—N1—Cu1	114.11 (16)	O1—C11—C11^i^	119.6 (2)
C10—N2—C6	118.8 (2)	N3—C11—C11^i^	112.8 (2)
C10—N2—Cu1	126.04 (16)	S1—C12—H12A	109.5
C6—N2—Cu1	114.74 (16)	S1—C12—H12B	109.5
C11—N3—O5	116.51 (19)	H12A—C12—H12B	109.5
C11—N3—Cu1^i^	114.16 (16)	S1—C12—H12C	109.5
O5—N3—Cu1^i^	129.32 (14)	H12A—C12—H12C	109.5
N1—C1—C2	122.0 (2)	H12B—C12—H12C	109.5
N1—C1—H1	119.0	S1—C13—H13A	109.5
C2—C1—H1	119.0	S1—C13—H13B	109.5
C3—C2—C1	119.3 (2)	H13A—C13—H13B	109.5
C3—C2—H2	120.4	S1—C13—H13C	109.5
C1—C2—H2	120.4	H13A—C13—H13C	109.5
C2—C3—C4	119.2 (2)	H13B—C13—H13C	109.5

Symmetry code: (i) −*x*+1, −*y*, −*z*+1.

### Hydrogen-bond geometry (Å, º)

**Table:**

*D*—H···*A*	*D*—H	H···*A*	*D*···*A*	*D*—H···*A*
O5—H5*O*···O6	0.92 (5)	2.12 (5)	2.912 (3)	144 (4)
C4—H4···O2^ii^	0.95	2.42	3.359 (3)	171
C7—H7···O2^ii^	0.95	2.31	3.226 (3)	162
C3—H3···O7^iii^	0.95	2.50	3.239 (3)	134
C13—H13*A*···O7^iv^	0.98	2.56	3.409 (3)	145
C13—H13*C*···O8^v^	0.98	2.48	3.346 (3)	148
C13—H13*B*···O8^vi^	0.98	2.65	3.442 (3)	138
C12—H12*A*···O9	0.98	2.36	3.175 (3)	140
C8—H8···O9^ii^	0.95	2.56	3.462 (3)	159
C12—H12*B*···O9^vi^	0.98	2.59	3.470 (3)	150

Symmetry codes: (ii) *x*+1, *y*, *z*; (iii) *x*+1, *y*+1, *z*; (iv) −*x*+1, −*y*, −*z*; (v) *x*, *y*+1, *z*; (vi) −*x*+2, −*y*, −*z*.
